# Multi-Toxin Resistance Enables Pink Bollworm Survival on Pyramided Bt Cotton

**DOI:** 10.1038/srep16554

**Published:** 2015-11-12

**Authors:** Jeffrey A. Fabrick, Gopalan C. Unnithan, Alex J. Yelich, Ben DeGain, Luke Masson, Jie Zhang, Yves Carrière, Bruce E. Tabashnik

**Affiliations:** 1 USDA ARS, U.S. Arid Land Agricultural Research Center, Maricopa, AZ 85138 USA; 2 Department of Entomology, University of Arizona, Tucson, AZ 85721 USA; 3 Biotechnology Research Institute, National Research Council of Canada, Montreal, QC, Canada H4P 2R2; 4 State Key Laboratory for Biology of Plant Diseases and Insect Pests, Institute of Plant Protection, Chinese Academy of Agricultural Sciences, Beijing, Haidian District, 100193 Peoples Republic of China

## Abstract

Transgenic crops producing *Bacillus thuringiensis* (Bt) proteins kill key insect pests, providing economic and environmental benefits. However, the evolution of pest resistance threatens the continued success of such Bt crops. To delay or counter resistance, transgenic plant “pyramids” producing two or more Bt proteins that kill the same pest have been adopted extensively. Field populations of the pink bollworm (*Pectinophora gossypiella*) in the United States have remained susceptible to Bt toxins Cry1Ac and Cry2Ab, but field-evolved practical resistance to Bt cotton producing Cry1Ac has occurred widely in India. Here we used two rounds of laboratory selection to achieve 18,000- to 150,000-fold resistance to Cry2Ab in pink bollworm. Inheritance of resistance to Cry2Ab was recessive, autosomal, conferred primarily by one locus, and independent of Cry1Ac resistance. We created a strain with high resistance to both toxins by crossing the Cry2Ab-resistant strain with a Cry1Ac-resistant strain, followed by one selection with Cry2Ab. This multi-toxin resistant strain survived on field-collected Bt cotton bolls producing both toxins. The results here demonstrate the risk of evolution of resistance to pyramided Bt plants, particularly when toxins are deployed sequentially and refuges are scarce, as seen with Bt cotton and pink bollworm in India.

Insecticidal crystalline proteins from the bacterium *Bacillus thuringiensis* (Bt) kill some key pests, but are harmless to most non-target organisms including people^1^,^2^,^3^,^4^. In 2014, the area planted worldwide to genetically engineered cotton, corn and soybean producing Bt proteins grew to 78 million hectares (ha)^5^. Although Bt crops can increase yield and profit, suppress pests, and decrease reliance on conventional insecticides^6^,^7^,^8^,^9^,^10^, evolution of resistance by insect pests can reduce these benefits^11^.

To delay or counter resistance to Bt crops, many growers have switched from transgenic plants that produce one Bt toxin to those producing two or more Bt toxins that kill the same pest^12^. These combinations of toxins called “pyramids” are especially effective when insects resistant to one toxin are killed by another toxin produced by the same plant^13^,^14^. Two conditions favoring success of two-toxin pyramids are: 1) pests are susceptible to both toxins and 2) resistance to one toxin does not cause cross-resistance to the other toxin^12^.

The most widely used pyramid of Bt cotton (*Gossypium hirsutum* L.) produces Bt toxins Cry1Ac and Cry2Ab. These toxins bind to different receptors in the midgut of lepidopteran larvae^15^,^16^ and cross-resistance between them is usually weak or nil^12^. In the United States, this two-toxin cotton was first planted in 2003 and reached 3.5 million ha by 2012, equivalent to 69% of the nation’s cotton^17^,^18^. Farmers in India first planted this two-toxin cotton in 2006, and its area increased to 11 million ha by 2013, accounting for 91% of India’s cotton^19^.

The lepidopteran pests targeted by Bt cotton include the pink bollworm, *Pectinophora gossypiella* (Saunders), which has been a major pest in many countries including the world’s three leading cotton producers (China, India, and the United States)^20^. In the United States, refuges of non-Bt cotton and mass releases of sterile moths have sustained pink bollworm susceptibility to Bt toxins for two decades, helping to bring this invasive insect close to eradication in Arizona and other southwestern states^9^,^21^. In India, because farmers have not planted adequate refuges, the efficacy of Bt cotton producing Cry1Ac has been eroded by widespread, field-evolved practical resistance^22^,^23^,^24^,^25^,^26^,^27^. In China, where farmers have not switched to two-toxin Bt cotton, small but significant increases in pink bollworm resistance to Cry1Ac have occurred in northern China, where close to 100% of the three million ha of cotton planted yearly produces Cry1Ac as the only Bt toxin^28^,^29^. As far as we know, field-evolved resistance to Cry2Ab has not been documented in pink bollworm.

Here we analyzed eight strains of pink bollworm from Arizona (Table 1) to improve our understanding of resistance to Cry1Ac and Cry2Ab in this global pest. In previous work, laboratory selection with either Cry1Ac only or both Cry1Ac and Cry2Ab yielded pink bollworm strains that survived on Bt cotton producing Cry1Ac, but not on Bt cotton producing both toxins^30^,^31^. Whereas some of these strains had >1000-fold resistance to Cry1Ac, the highest previously reported pink bollworm resistance to Cry2Ab was 240-fold^11^,^31^. In this study, two rounds of laboratory selection with Cry2Ab generated 18,000- to 150,000-fold resistance to Cry2Ab in a strain named Bt4-R2. After we crossed Bt4-R2 with a Cry1Ac-resistant strain (AZP-R)^30^,^32^ and selected once on diet with Cry2Ab, the resulting multi-toxin resistant strain (AZP-R2) survived on bolls of Bt cotton producing both toxins. We used Bt4R-2, AZP-R2 and other strains to assess inheritance and fitness costs of resistance to Cry2Ab, as well as cross-resistance between Cry1Ac and Cry2Ab.

## Results

### Selection for resistance to Cry2Ab in strain Bt4-R2

**Figure 1:**
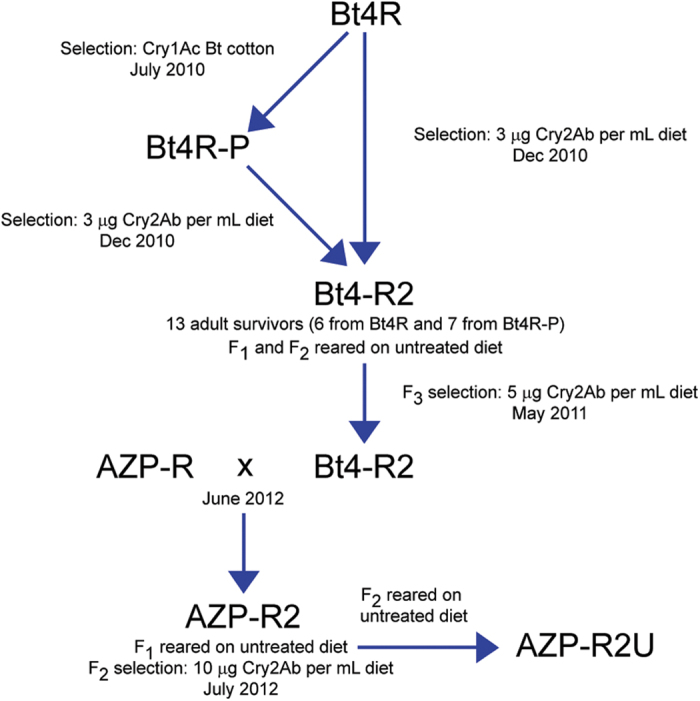
Selection for pink bollworm resistance to Cry1Ac and Cry2Ab. We created strain Bt4R-P by selecting a subset of larvae from the Cry1Ac-resistant strain Bt4R on bolls of Bt cotton producing Cry1Ac. Next we exposed a total of 10,000 to 20,000 neonates from Bt4R and Bt4R-P to diet containing 3 μg Cry2Ab per mL diet. We started the Cry2Ab-resistant strain Bt4-R2 by pooling the adult survivors from Bt4R (n = 6) and Bt4R-P (n = 7). These 13 adults mated and produced eggs. The next two generations were reared on untreated diet. The F_3_ larvae of the Bt4-R2 strain were reared on diet containing 5 μg Cry2Ab per mL diet, yielding extremely high resistance to Cry2Ab (Fig. 2 and Tables 2 and 3). We crossed AZP-R (highly resistant to Cry1Ac) with Bt4-R2 to start the AZP-R2 strain. We selected F_2_ larvae of AZP-R2 on diet containing 10 μg Cry2Ab per mL diet, which yielded high, nearly homogeneous resistance to both Cry1Ac and Cry2Ab in the F_4_ larvae of AZP-R2 (Table 4). AZP-R2U was a subset of AZP-R2 that was reared without additional selection on Cry2Ab.

The Cry2Ab-resistant Bt4-R2 strain was derived from the Cry1Ac-resistant strain Bt4R^33^ (Fig. 1). Before selection for resistance to Cry2Ab, a subset of Bt4R, designated Bt4R-P, was selected on cotton plants producing Cry1Ac.

In the first selection with Cry2Ab, we exposed approximately 5,000 to 10,000 neonates from Bt4R and a similar number from Bt4R-P to diet containing 3 μg Cry2Ab per mL diet. This yielded six survivors from Bt4R and seven from Bt4R-P for a total of 13 survivors from the 10,000 to 20,000 neonates exposed (ca. 0.1% survival). These 13 survivors mated among themselves to start the Bt4-R2 strain. After rearing Bt4-R2 on untreated diet for two generations (F_1_ and F_2_), we conducted the second round of selection by exposing approximately 20,000 to 30,000 neonates of the F_3_ generation to diet containing 5 μg Cry2Ab per mL, which yielded 877 survivors (ca. 3% survival).

**Figure 2:**
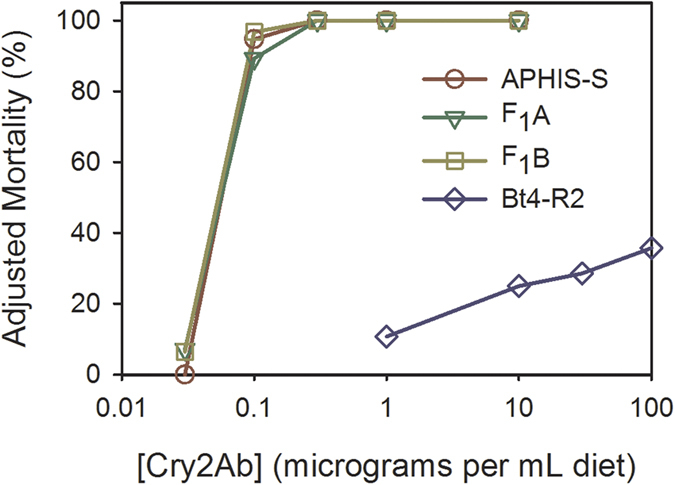
Responses to Cry2Ab of pink bollworm larvae from a susceptible strain (APHIS-S), a resistant strain (Bt4-R2), and their F_1_ progeny. We scored live fourth instars, pupae, and adults as survivors after 21 d on diet. Adjusted mortality (%) is 100% minus adjusted survival (%). Adjusted survival (%) is survival on Cry2Ab-treated diet divided by survival on untreated control diet multiplied by 100%. F_1_A and F_1_B indicate F_1_ progeny from mass crosses with male Bt4-R2 × female APHIS-S and female Bt4-R2 × male APHIS-S, respectively.

**Figure 3:**
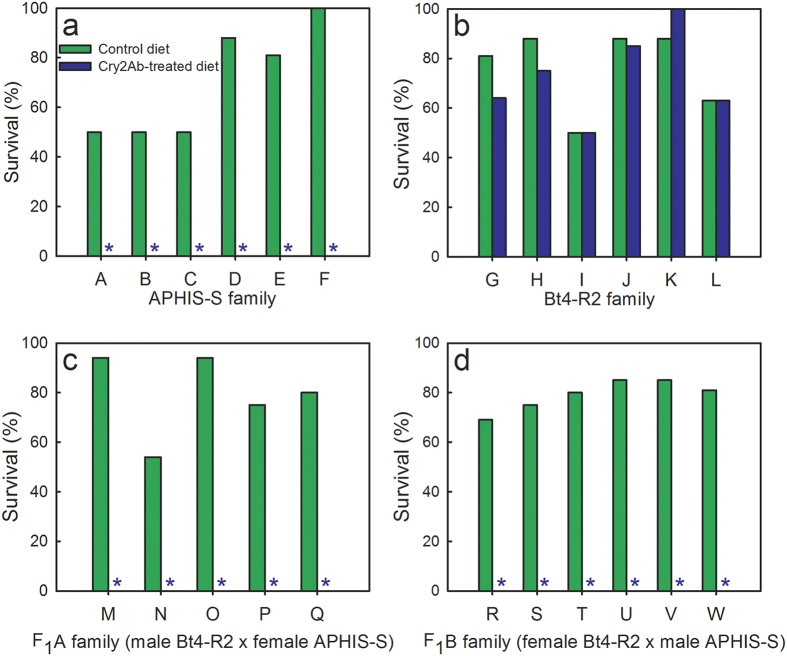
Responses to Cry2Ab of pink bollworm larvae from single-pair families from a susceptible strain (APHIS-S) (a), a resistant strain (Bt4-R2) (b), and their F_1_ progeny (**c, d**). Survival (%) on control diet (no Bt toxin, green bars) and diet treated with 10 μg Cry2Ab per mL diet (blue bars). Each of the 23 families tested (A-W) was generated by crossing a single male with a single female (n = 8–24 larvae tested per family on each diet; mean = 16 neonates per family on each diet). Asterisks indicate 0% survival on Cry2Ab-treated diet for all 17 families from APHIS-S and F_1_.

These two rounds of selection with Cry2Ab generated extremely high resistance to Cry2Ab in Bt4-R2 (Tables 2 and 3, Figs 2 and 3). After these two rounds of selection with Cry2Ab, Bt4-R2 was reared without additional exposure to any Bt toxins for the remainder of this study. We calculated the resistance ratio as the concentration of toxin killing 50% of larvae (LC_50_) for a strain divided by the LC_50_ for the susceptible strain APHIS-S tested during the same time period. The resistance ratio for Bt4-R2 to Cry2Ab was 150,000 for the F_15_ generation and 18,000 for the F_22_ generation (Tables 2 and 3). The highest concentration of Cry2Ab tested, 600 μg Cry2Ab per mL diet, killed only 23% of larvae from the F_15_ generation of Bt4-R2 (n = 30). Because it was difficult to kill Bt4-R2 with the highest concentrations of Cry2Ab tested, we consider the resistance ratios approximations, and cannot determine if the difference in LC_50_ between the F_15_ and F_22_ generations is statistically significant. At the diagnostic concentration of 10 μg Cry2Ab per mL diet, adjusted larval survival ranged from 75 to 100% for Bt4-R2 compared with 0% for all other strains tested (Tables 2 and 3, Figs 2 and 3). The LC_50_ of Cry2Ab did not differ significantly among Bt4R, Bt4R-P and APHIS-S, indicating that both parent strains of Bt4-R2 were susceptible to Cry2Ab (Table 2).

### Evaluation of cross-resistance between Cry1Ac and Cry2Ab in Bt4-R2 and its parent strains

The results show little or no cross-resistance occurred between Cry1Ac and Cry2Ab in Bt4-R2 and its parent strains (Table 2). The LC_50_ of Cry1Ac did not differ significantly between Bt4-R2 and Bt4R, and was significantly less for Bt4-R2 than Bt4R-P (Table 2), which indicates that selection with Cry2Ab did not increase resistance to Cry1Ac of Bt4-R2 relative to its parent strains. In addition, selection with Cry1Ac increased the resistance ratio for Cry1Ac to 400 in Bt4R-P without increasing its resistance to Cry2Ab (resistance ratio = 0.58).

### Inheritance of resistance to Cry2Ab in strain Bt4-R2

To evaluate inheritance of resistance to Cry2Ab, we used diet bioassays to test Bt4-R2, APHIS-S, and their F_1_ progeny. We generated the F_1_ progeny with two types of reciprocal crosses: mass crosses and single-pair crosses. Responses of the F_1_ progeny from both sets of crosses show that the resistance to Cry2Ab in Bt4-R2 was autosomal and recessive (Table 3 and Figs 2 and 3).

#### Mass crosses

In the mass cross experiment, the LC_50_ was similar for the progeny from the two reciprocal crosses (Table 3), indicating we did not detect maternal effects or sex linkage, which shows that the inheritance of resistance to Cry2Ab in Bt4-R2 is autosomal. For the F_1_ pooled from the two reciprocal mass crosses, the LC_50_ did not differ significantly from the LC_50_ of the susceptible APHIS-S strain (Table 3), indicating recessive inheritance of resistance. The resistance ratio was 1.6 for the F_1_ pooled from the mass crosses compared with 18,000 for the F_22_ of Bt4-R2 used as parents in the reciprocal crosses.

We calculated the dominance parameter *h*, which varies from zero for completely recessive resistance to one for completely dominant resistance. For the F_1_ pooled from the mass crosses, survival was 0% yielding *h* = 0 at both concentrations that were tested against the F_1_, Bt4-R2 and APHIS-S (1 and 10 μg Cry1Ac per mL diet, Fig. 2).

#### Single-pair crosses

The results from the single-pair crosses confirm that inheritance of resistance to Cry2Ab was autosomal and recessive (Fig. 3). At the diagnostic concentration of Cry2Ab (10 μg Cry2Ab per mL diet), adjusted survival was 0% for all 11 F_1_ families, 0% for all six families from APHIS-S, and 79 to 100% for six families from Bt4-R2 (Fig. 3). These results yield *h* = 0 for all 11 F_1_ families tested, indicating completely recessive inheritance at the diagnostic concentration of Cry2Ab.

### AZP-R crossed with Bt4-R2 to create multi-toxin resistant strain AZP-R2

Although the Bt4-R2 strain was extremely resistant to Cry2Ab, it had only 28-fold resistance to Cry1Ac relative to the susceptible APHIS-S strain (Table 2). Conversely, the AZP-R strain had 1,500-fold resistance to Cry1Ac, but only two-fold resistance to Cry2Ab^31^. To generate a strain with high resistance to both Cry1Ac and Cry2Ab, we crossed AZP-R with Bt4-R2 to create the AZP-R2 strain. To start the AZP-R2 strain, we used two replicates of 50 female pupae from Bt4-R2 pooled with 50 male pupae from AZP-R and two replicates of 50 female pupae from AZP-R pooled with 50 male pupae from Bt4-R2 (total n = 400 pupae). After the adults mated, the F_1_ eggs from all four replicates were combined as AZP-R2 and the resulting larvae were reared on untreated diet.

### Inheritance of resistance to Cry2Ab in AZP-R2

We hypothesized that the resistance to Cry2Ab in AZP-R2 is recessive, because it was derived from the recessive resistance to Cry2Ab in Bt4-R2. Given that AZP-R was not resistant to Cry2Ab, we hypothesized the F_1_ offspring of the cross between AZP-R and Bt4-R2 would be heterozygous for Cry2Ab resistance, with a recessive resistance allele from Bt4-R2 and a susceptible allele from AZP-R, yielding no resistant individuals. Consistent with this prediction, adjusted survival of the F_1_ larvae of AZP-R2 was 0% at a diagnostic concentration of Cry2Ab (Table 4).

If the resistance in AZP-R2 is conferred by a recessive allele at one locus, the expected survival of the F_2_ of AZP-R2 at a diagnostic concentration of Cry2Ab is 25% based on Mendelian inheritance (i.e., 25% resistant homozygotes). Consistent with this prediction, the adjusted survival of F_2_ larvae from AZP-R2 at the diagnostic concentration of Cry2Ab was 25% (Table 4). We exposed 1,020 F_2_ larvae of AZP-R2 to the diagnostic concentration of Cry2Ab and reared the progeny of the survivors (F_3_) on untreated diet. Adjusted survival of the F_4_ larvae at the diagnostic concentration of Cry2Ab was 100% (n = 80 larvae, 40 on treated diet and 40 on untreated diet), which is consistent with the expectation that the single selection allowed survival only of the individuals with homozygous resistance to Cry2Ab.

### Inheritance of resistance to Cry1Ac in AZP-R2

We hypothesized that the resistance to Cry1Ac in AZP-R2 is conferred by recessive alleles at the cadherin locus *PgCad1* because previous work showed that recessive alleles at this locus confer resistance to Cry1Ac in both AZP-R and Bt4R^33^,^34^. Previous results also showed that crosses between homozygous Cry1Ac-resistant individuals from these two strains yielded offspring with 100% survival at the diagnostic concentration of Cry1Ac (10 μg Cry1Ac per mL diet)^33^. Given that resistance of Bt4-R2 to Cry1Ac was derived from Bt4R, we expected that a cross between Cry1Ac-resistant adults from Bt4R-2 and AZP-R would yield AZP-R2 offspring resistant to Cry1Ac. When Bt4-R2 and AZP-R were crossed to generate AZP-R2, concurrent bioassays showed that survival at the diagnostic concentration was 100% for AZP-R and 86.2% for Bt4-R2 (n = 60 larvae per strain, 30 tested at the diagnostic concentration and 30 on control diet). The higher survival of Bt4-R2 in this test conducted at the University of Arizona compared with the 56.5% survival when Bt4-R2 was tested previously at USDA ARS U.S. Arid Land Agricultural Research Center (ALARC) (Table 2) could reflect differences between the two laboratories in bioassay methods, changes in the strain over time, or both.

Assuming Hardy-Weinberg equilibrium and recessive inheritance of resistance conferred by the cadherin locus, the estimated genotype frequency at the cadherin locus when AZP-R2 was generated is 1.0 resistant homozygotes for AZP-R, and 0.862 resistant homozygotes and 0.133 heterozygotes for Bt4-R2. Based on these frequencies, we predicted survival of the F_1_ and subsequent generations of AZP-R2 at the diagnostic concentration of Cry1Ac would be 93% (0.862 + 0.5[0.133] = 0.928). The mean adjusted survival of F_1_, F_2_ and F_4_ larvae of AZP-R2 was 95% (range = 90 to 100%) (Table 4), which does not differ significantly from the predicted 93% (one-sample t-test, t = 0.69, P = 0.56).

In AZP-R2, the percentage of resistant individuals was 90% in the F_1_ and 100% in the F_2_ for Cry1Ac, compared with 0% in the F_1_ and 25% in the F_2_ for Cry2Ab (Table 4). The lower resistance to Cry2Ab than Cry1Ac in these two generations indicates that the cadherin alleles conferring resistance to Cry1Ac in AZP-R2 conferred little or no cross-resistance to Cry2Ab.

### Survival and development rate on Bt cotton bolls producing Cry1Ac and Cry2Ab

**Figure 4:**
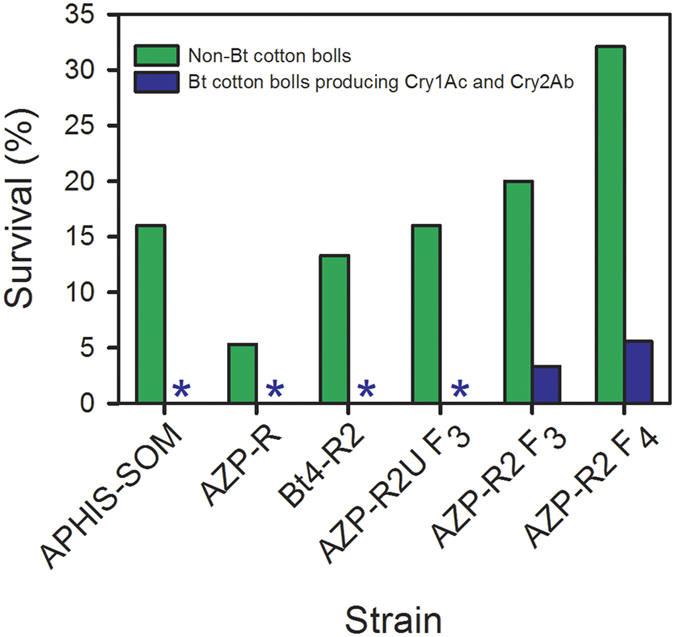
Survival of pink bollworm larvae on field-grown cotton bolls. Strains were susceptible (APHIS-SOM), resistant to Cry1Ac (AZP-R), moderately resistant to Cry1Ac and highly resistant to Cry2Ab (Bt4-R2), uniformly resistant to Cry1Ac and 25% of larvae resistant to Cry2Ab (AZP-R2U F_3_), and highly resistant to Cry1Ac and Cry2Ab (AZP-R2 F_3_ and F_4_). Survival in the laboratory on bolls collected from field-grown plants of non-Bt cotton (green) and Bt cotton producing Cry1Ac and Cry2Ab (blue). Asterisks indicate 0% survival on Bt cotton bolls producing Cry1Ac and Cry2Ab for all larvae tested except AZP-R2 F_3_ and F_4_, which both had survival of 0.17 on two-toxin cotton relative to non-Bt cotton (see Table S1 and Methods for details).

The only strain with survivors on field-collected bolls of two-toxin cotton was AZP-R2, which had survivors in both generations tested (F_3_ = 3.3% and F_4_ = 5.6%, Fig. 4 and Table S1). Survival of larvae reared on field-collected bolls producing Cry1Ac and Cry2Ab was 0% for the other four strains tested (Fig. 4 and Table S1): susceptible strain APHIS-SOM, Cry1Ac-resistant strain AZP-R, Bt4-R2, which had moderate resistance to Cry1Ac and high resistance to Cry2Ab (Tables 2 and 3), and AZP-R2U, a subset of AZP-R2 that was not selected with Cry2Ab. Survival on two-toxin bolls was higher for AZP-R2 than AZP-R2U (Fisher’s exact test, total n = 749, P = 0.002), which is consistent with the diet bioassay results (Table 4) indicating that selection of the F_2_ with Cry2Ab increased the percentage of individuals resistant to Cry2Ab in AZP-R2.

Relative to survival on field-collected bolls of non-Bt cotton, the proportion of larval survival on bolls producing both toxins was 0.17 for both F_3_ and F_4_ generations of AZP-R2 (Fig. 4 and Table S1), indicating incomplete resistance^35^. For AZP-R2, the percentage of survivors reaching pupation by the end of the bioassays was 0% on two-toxin bolls versus 93% on non-Bt bolls in the F_3_ (Fisher’s exact test, total n = 20, P = 0.0004) and 8.0% on two-toxin bolls versus 69% on non-Bt bolls in the F_4_ (Fisher’s exact test, total n = 51, P < 0.0001). The slower development of AZP-R2 on two-toxin bolls relative to non-Bt cotton bolls also indicates incomplete resistance.

### Evaluation of fitness costs associated with increased survival on two-toxin Bt cotton

To check for fitness costs, we compared performance on non-Bt cotton bolls for the F_3_ and F_4_ of AZP-R2 (which both had survival on two-toxin bolls) relative to AZP-R2U, as well as APHIS-SOM, AZP-R, Bt4-R2 (which all had 0% survival on two-toxin bolls). The specific comparison between the F_3_ of AZP-R2 and AZP-R2U is especially relevant because these two sets of larvae were tested simultaneously, had a similar genetic background, and differed only in that the parents of AZP-R2, but not AZP-R2U, were selected with Cry2Ab. Larval survival on non-Bt cotton bolls for the F_3_ was not lower for AZP-R2 (20%) than AZP-R2U (16%) (Fig. 4 and Table S1). These results imply that a fitness cost affecting survival on non-Bt cotton was not associated with the increased resistance to Cry2Ab and increased survival on two-toxin bolls of AZP-R2 versus AZP-R2U. Larval survival on non-Bt cotton bolls was not lower for the F_3_ and F_4_ of AZP-R2 relative to any of the other strains tested (Fig. 4 and Table S1), yet these more general comparisons may be affected by differences between strains that are not directly related to resistance.

The percentage of F_3_ survivors reaching pupation after 21 days on non-Bt bolls was not lower for AZP-R2 (93%) than AZP-R2U (92%). As described above for survival on non-Bt bolls, these results indicate that no major fitness cost affecting the percentage of survivors pupating after 21 days on non-Bt bolls was associated with the increased resistance to Cry2Ab of AZP-R2 relative to AZP-R2U. The percentage of F_3_ survivors that were adults after 21 days on non-Bt bolls was 0% for AZP-R2 versus 17% for AZP-R2U, but this difference suggesting a potential fitness cost is not statistically significant (Fisher’s exact test, total n = 27, P = 0.19). In addition, the percentage of survivors that were adults after 21 days on non-Bt bolls did not differ significantly between the F_3_ and F_4_ of AZP-R2 pooled (0%) and the four other strains pooled (7.9% for APHIS-SOM, AZP-R, Bt4-R2 and AZP-R2U; Fisher’s exact test, total n = 79, P = 0.11). Nonetheless, more extensive testing might reveal fitness costs affecting development rate or other traits.

## Discussion

We started with pink bollworm strains that were susceptible to Cry2Ab and achieved 18,000- to 150,000-fold resistance to Cry2Ab in strain Bt4-R2 after only two generations of laboratory selection (Fig. 1, Tables 2 and 3). To create the AZP-R2 strain with high levels of resistance to both toxins, we crossed Bt4R-2 with the AZP-R strain, which was highly resistant to Cry1Ac but not to Cry2Ab^31^. The results indicate inheritance of resistance to Cry2Ab in Bt4-R2 and AZP-R2 is autosomal, recessive, and probably conferred by a single locus (Figs 2 and 3, Tables 3 and 4). In boll bioassays conducted after one additional selection of AZP-R2 with Cry2Ab, the proportion of larval survival on Bt cotton producing Cry1Ac and Cry2Ab relative to non-Bt cotton was 0.17 for AZP-R2 (Fig. 4 and Table S1). The lower survival and slower development of AZP-R2 on two-toxin cotton relative to non-Bt cotton indicate incomplete resistance^35^.

As far as we know, AZP-R2 is the first pink bollworm strain with documented survival on Bt cotton bolls producing both Cry1Ac and Cry2Ab. In the tests here, survival on two-toxin Bt cotton was 0% for AZP-R, AZP-R2U, and Bt4-R2 (Fig. 4). These results are consistent with previous results for AZP-R, indicating that larvae from this Cry1Ac-resistant strain were killed by the Cry2Ab in two-toxin cotton^31^,^36^. Analogously, we infer that the Cry2Ab in two-toxin Bt cotton killed larvae of AZP-R2U, a subset of AZP-R2 that was not selected with Cry2Ab after AZP-R2 was created. Conversely, the Cry1Ac in two-toxin cotton probably killed larvae of Bt4-R2, because it was highly resistant to Cry2Ab, but had only 28-fold resistance to Cry1Ac (Tables 2 and 3).

In previous work, pink bollworm strain BX-R1 had 0% survival on Bt cotton bolls producing Cry1Ac and Cry2Ab, despite its high survival on Bt cotton bolls producing Cry1Ac, 420-fold resistance to Cry1Ac, and 240-fold resistance to Cry2Ab^31^. Because the concentration of toxin is 160 times higher for Cry2Ab than Cry1Ac in bolls of two-toxin cotton^37^, the minimum LC_50_ value required for survival on these bolls is expected to be much higher for Cry2Ab than Cry1Ac. We conclude that survival on two-toxin cotton bolls was higher for AZP-R2 than BX-R1 primarily because of the higher resistance of AZP-R2 to Cry2Ab.

With some assumptions, we can estimate the initial frequency of Cry2Ab resistance alleles in Bt4R-P and Bt4R. If 1 to 13 of the survivors from the approximately15,000 larvae screened initially with Cry2Ab were homozygous resistant, the frequency of homozygous resistant individuals in these strains was roughly 0.00007 to 0.0009. Assuming Hardy-Weinberg equilibrium, the estimated initial Cry2Ab resistance allele frequency is about 0.008 to 0.03. The lower limit is almost certainly an underestimate, because it is unlikely that just one of the 13 survivors of the initial selection was resistant to Cry2Ab. Although alleles conferring high resistance to Cry2Ab were not extremely rare in the Bt4R and Bt4R-P strains, such alleles were not detected in other strains of pink bollworm, including the BX-R1, BX-R2 and BX-R strains that were selected extensively with Cry2Ab^31^. For comparison, the estimated Cry1Ac resistance allele frequency for Arizona field populations of pink bollworm was 0.16 in 1997, the second year of Bt cotton cultivation^32^. The estimated Cry2Ab resistance allele frequency for *Helicoverpa punctigera* in Australia was 0.006 in non-cropping areas and 0.008 in cropping areas before cotton producing Cry2Ab was commercialized^38^.

Consistent with previous results from lab- and field-selected strains of pink bollworm from Arizona and India, respectively^22^,^25^,^26^,^30^,^31^, the results here show that pink bollworm resistance to Cry1Ac does not confer strong cross-resistance to Cry2Ab. For example, Bt4R-P had 400-fold resistance to Cry1Ac, yet was susceptible to Cry2Ab (Table 2). Also, survival at a diagnostic toxin concentration for the F_1_ generation of AZP-R2 was 90% for Cry1Ac and 0% for Cry2Ab (Table 4).

We also found here that selection for resistance to Cry2Ab did not cause strong cross-resistance to Cry1Ac. For example, relative to its parent strains Bt4R and Bt4R-P, selection of Bt4R-2 with Cry2Ab increased resistance to Cry2Ab >150,000-fold, but resistance of Bt4R-2 to Cry1Ac was only 28-fold, which is between the resistance to Cry1Ac of its two parent strains (11- and 400-fold for Bt4R and Bt4R-P, respectively, Table 2). This pattern differs from our previous results with the BX-R1 and BX-R2 strains of pink bollworm, where selection with Cry2Ab produced up to 420-fold cross-resistance to Cry1Ac^31^. While the mechanism of the previously reported “asymmetrical” cross-resistance between Cry2Ab and Cry1Ac remains unknown, the results here and previously^31^,^33^ demonstrate that the pink bollworm mutations conferring resistance to Cry1Ac by disrupting a Cry1Ac-binding cadherin protein do not confer cross-resistance to Cry2Ab. Furthermore, given that Cry1A and Cry2A bind to different sites in the larval midgut of pink bollworm^15^,^16^, it is unlikely that mutations affecting a single toxin-binding protein can confer resistance to both toxins in this pest^31^. In *Spodoptera exigua*, however, both Cry1Ac and Cry2Ab bind to midgut cadherin^39^.

We previously hypothesized that two or more loci confer resistance to Cry2Ab in the BX-R strains^31^. If true, this would differ from the apparent single-locus control of resistance to Cry2Ab in Bt4-R2 and AZP-R2 seen here. Because resistance to Cry1Ac is recessive in the BX-R strains^31^ as well as in AZP-R2 and Bt4R-2, interstrain complementation tests should be useful for determining if the Cry2Ab resistance alleles in the BX-R strains and those found here occur at the same locus.

Although survival on Bt cotton producing Cry1Ac and Cry2Ab was not reported previously for pink bollworm, it is documented for two other major lepidopteran pests: *Helicoverpa zea* in independent field, greenhouse and laboratory experiments^17^,^40^,^41^ and *Trichoplusia ni* reared for seven days in the laboratory on cotton leaves, then transferred to whole cotton plants in the greenhouse^42^. The results with *T. ni* are strikingly similar to the results here with pink bollworm, including high levels of resistance to both toxins, incomplete resistance to two-toxin Bt cotton, little or no cross-resistance between the two toxins, and autosomal, recessive inheritance to each toxin conferred by a different locus^42^,^43^. The resistance of *T. ni* to Cry2Ab was not linked with cadherin, nor several other candidate resistance genes including the ATP-binding cassette transporter gene *ABCC2*, which is linked with resistance to Cry1A toxins in *T. ni* and several other lepidopterans^44^,^45^.

Although incomplete resistance to two-toxin Bt cotton was also seen in the GA-R strain of *H. zea*^17^, other aspects of its resistance differ markedly from those of pink bollworm and *T. ni*, including non-recessive inheritance of resistance to Cry1Ac, less than five-fold resistance to Cry2Ab, and selection with Cry1Ac that increased survival on two-toxin cotton^17^. Direct interspecific comparisons between susceptible strains show that intrinsic susceptibility is much lower for *H. zea* than pink bollworm for both Cry1Ac (72-fold) and Cry2Ab (485-fold)^37^. Field-evolved resistance has been documented in populations of *H. zea, T. ni*, and pink bollworm to Cry1Ac, but only for *H. zea* to Cry2Ab^11^,^22^,^23^,^24^,^25^,^26^,^45^,^46^,^47^.

The pink bollworm survival on Bt cotton producing Cry1Ac and Cry2Ab reported here has implications for managing this pest in the field, particularly in China and India. Given that our multi-toxin resistant strain was generated from relatively small populations selected in the laboratory, the billions of pink bollworm larvae in field populations exposed to Bt cotton in China and India are also expected to harbor alleles for resistance to both toxins. Indeed, pink bollworm populations in India have diverse mutations in cadherin genes associated with field-evolved resistance to Bt cotton producing Cry1Ac^23^. The risk of resistance is high in both countries, because neither has abundant refuges of non-Bt cotton or other pink bollworm host plants^24^,^25^,^26^,^28^. For populations resistant to Cry1Ac, only the Cry2Ab in two-toxin cotton is effective. In northern China, where a low, but significant increase in the percentage of pink bollworm resistant to Cry1Ac has been reported^28^, the two-toxin cotton is likely to be more durable if it is adopted before resistance to Cry1Ac becomes more common. In India, where resistance to Cry1Ac is already widespread and grower compliance with the refuge strategy is low, we expect rapid evolution of resistance to Cry2Ab. The genetically modified toxins Cry1AbMod and Cry1AcMod were effective in the laboratory against pink bollworm with resistance to Cry1Ac and Cry2Ab^11^, yet their efficacy in the field remains to be tested.

## Methods

### Insects

We used eight strains of pink bollworm from Arizona: APHIS-S, APHIS-SOM, Bt4R, Bt4R-P, Bt4-R2, AZP-R, AZP-R2, and AZP-R2U (Table 1 and Fig. 1). APHIS-S is a susceptible strain that had been reared in the laboratory for more than 30 years without exposure to Bt toxins^49^,^50^. APHIS-SOM is a susceptible strain derived by crossing APHIS-S with a susceptible field strain, collected from Somerton, Arizona in 2007^33^. Bt4R is a laboratory-selected strain with moderate resistance to Cry1Ac and previously demonstrated survival on Bt cotton producing Cry1Ac Bt^33^,^51^,^52^. Bt4R-P was derived from Bt4R by greenhouse selection of larvae on Bollgard® DP 449 BG/RR cotton bolls in July 2010 (Fig. 1). Survivors of selection on 3 μg Cry2Ab per mL diet from both Bt4R (n = 6) and Bt4R-P (n = 7) were pooled as pupae and eggs collected from adults were used to start the Bt4-R2 strain in Dec. 2010 (Fig. 1). A second selection of Bt4-R2 on 5 μg Cry2Ab per mL diet was done before performing concentration-response and crossing experiments. AZP-R is a Cry1Ac-resistant strain that was started by pooling survivors of exposure to Cry1Ac in diet from 10 populations derived in 1997 from Arizona cotton fields^53^. In June 2012, we crossed Bt4-R2 with AZP-R to create the AZP-R2 strain (Fig. 1). We selected the F_2_ progeny of AZP-R2 on diet with 10 μg Cry2Ab per mL (Fig. 1). The crosses to generate AZP-R2 and all subsequent experiments were conducted at the University of Arizona in Tucson. All other experiments were conducted at the USDA ARS U.S. ALARC in Maricopa, Arizona.

### Diet Bioassays

We used 21-d diet incorporation bioassays to evaluate susceptibility to Cry1Ac and Cry2Ab^31^,^33^,^36^,^53^. Newly hatched neonates were placed individually in wells of bioassay trays (BIO-BA-128, Pitman, NJ) containing approximately 1.5 mL of diet and covered with Pull N’ Peel tab tray covers (BIO-CU-16, Pitman, NJ). We tested five to eight concentrations of each toxin ranging from 0–1,000 μg toxin per mL diet. After 21 d at 26 °C and a photoperiod of 14 light:10 dark, we scored live fourth instar larvae, pupae, and adults as survivors.

### Bt Toxins

The source of Cry1Ac was MVP II (Dow Agrosciences, San Diego, CA), a liquid formulation containing a hybrid protoxin produced in and encapsulated by *Pseudomonas fluorescens*^30^,^54^. The protoxin amino sequence is 98.5% identical between MVP II and Cry1Ac. The first 1,067 of 1,182 amino acids in MVP II protoxin, including the entire active toxin (domains I, II, and III), are identical to the holotype Cry1Ac protoxin and are encoded by part of the Cry1Ac gene from *B. thuringiensis* subsp. *kurstaki* HD-73^54^. We used Cry2Ab protoxin from Luke Masson and Jie Zhang that was produced using a recombinant acrystalliferous strain of Bt subspecies *kurstaki* (HD73 *cry-*) that was transformed with the *cry2Ab* gene from strain HD1 of Bt subspecies *kurstaki* as described previously^55^,^56^. Although pink bollworm responses to Cry2Ab from the two sources were similar, we used Cry2Ab from one source for each experiment so that comparisons among strains and the progeny from crosses within experiments were not affected by potential differences between the two sources of Cry2Ab. One exception is the multi-generational data for AZP-R2 reported in Table 4, where the F_1_ was tested with Cry2Ab from Masson and the F_2_ and F_4_ were tested with Cry2Ab from Zhang.

### Mass crosses

We obtained and determined the sex of 120 Bt4-R2 pupae and 120 APHIS-S pupae and set up four reciprocal mass crosses including 30 ♂ Bt4-R2 × 30 ♀ APHIS-S, 30 ♀ Bt4-R2 × 30 ♂ APHIS-S, 30 ♀ APHIS-S × 30 ♂ APHIS-S, and 30 ♀ Bt4-R2 × 30 ♂ Bt4-R2 in paper cups (350 mL). Adults were allowed to mate and deposit eggs onto oviposition paper. Newly hatched F_1_ neonates were transferred to individual wells of bioassay trays containing various concentrations of Cry2Ab and maintained at 26 °C (14:10 L:D). After 21 days, live individuals that developed beyond the third instar were scored as survivors^49^. For all bioassays containing F_1_ neonates resulting from crosses for which at least one of the parents was APHIS-S, we used concentrations of 0, 0.03, 0.1, 0.3, 1, and 10 μg Cry2Ab per mL diet. Concentrations used in bioassays on F_1_ neonates resulting from the Bt4-R2 intrastrain mass cross were 0, 1, 10, 30, and 100 μg Cry2Ab per mL diet. We tested a total of 736 neonates including 32 neonates at each concentration for all four mass crosses.

### Single-pair crosses

To bolster evaluation of dominance, maternal effects and sex linkage, and to generate informative families for future biphasic genetic linkage analysis^34^,^57^, we performed single-pair reciprocal crosses between Bt4-R2 and APHIS-S. We obtained and tested progeny from five or six families from each of the four types of single-pair cross: ♂ Bt4-R2 × ♀ APHIS-S, ♀ Bt4-R2 × ♂ APHIS-S, ♀ APHIS-S × ♂ APHIS-S, and ♀ Bt4-R2 × ♂ Bt4-R2. The progeny from these crosses were tested for survival on control diet without any Bt toxin or on diet with 10 μg Cry2Ab per mL diet. After 14 days at 26 °C (14:10 L:D), we collected all surviving fourth instars and pupae.

### Boll bioassays

We evaluated survival and development rate in the laboratory using bolls collected from cotton planted on 23 April 2012 in heavy loam soil at the Campus Agricultural Center of the University of Arizona in Tucson, Arizona. Plants were grown using standard agronomic practices, flood irrigated, and not treated with insecticide. We used Bt cotton cultivar DP 164 B2RF, which produces Cry1Ac and Cry2Ab, and non-Bt cotton cultivar DP 5415.

We conducted two sets of boll bioassays. In the first set, we tested five strains: APHIS-SOM, AZP-R, Bt4-R2, AZP-R2, and AZP-R2U. In the second set, we tested only AZP-R2 to verify its survival on two-toxin Bt cotton that was seen in the first set. In the first set, bolls were collected on 20 August 2012 and infested on 22 August 2012. In the second set, bolls were collected and infested on 18 September 2012.

In both sets of bioassays, fruiting branches with bolls (about four to six weeks old) and leaves were cut from plants, put in plastic bags under cool conditions, and brought to the lab (6 km from the field). Bolls were briefly rinsed with dilute bleach (5%), washed with running water, and blotted dry. Leaves of plants from which the bolls were used were frozen for subsequent testing for Bt toxins (see below).

In the first assays, bolls with bracteoles removed were stored at 4 °C for two days before infestation. We put 15 neonates on the carpel surface of each boll using a fine brush. For each of the five strains tested, we infested 10 bolls of Bt cotton and five bolls of non-Bt cotton (total n = 75 bolls infested with 1,025 larvae). Before infestation, the mean fresh weight for the 75 bolls was 19 g (range = 15 to 23 g). We put each boll in a 150-mL plastic cup sealed with a plastic lid, and put the cups in an incubator at 29 °C (16:8 L:D). After two days, we loosened the lid, lined each cup with Kimwipe tissue paper, and transferred all cups to 1.5-L plastic buckets that were lined with paper towels and had lids with fine mesh windows. After two to four days in the buckets, lids were removed to increase ventilation and reduce mold growth. After 21 days, we cut bolls open and recorded survivors inside bolls and on the tissue paper in the buckets.

In the second assays, bolls were infested by putting a piece of paper towel (ca. 1 × 1 cm) bearing pink bollworm eggs under the bracteoles of each boll. We infested 30 Bt cotton bolls and 5 non-Bt cotton bolls using eggs from AZP-R2. We put each boll in a 150-mL plastic cup with a plastic lid and put the cups in an incubator at 29 °C (16:8 L:D). After two to four days, for each boll, we counted pink bollworm larval entry holes and removed the bracteoles and pieces of paper bearing eggs. We excluded 10 bolls of Bt cotton and one of non-Bt cotton from further consideration because mold prevented accurate identification of entry holes. For the remaining 20 bolls of Bt cotton and four bolls of non-Bt cotton, we transferred each boll to a new cup lined with tissue paper, and held the cups in the 1.5-L plastic buckets as described above. After 22–24 days, we checked for survivors as described above.

In both sets of bioassays, we scored live fourth instars and pupae as survivors. In the first set, we calculated survival as the number of survivors divided by the total number of larvae placed on the bolls. In the second set, we calculated survival as the number of survivors divided by the number of pink bollworm entry holes.

To check for Cry1Ac and Cry2Ab in leaves, we used the QuickStix Combo Kit for Cry1A and Cry2A Leaf & Seed (Envirologix, Portland, Maine) according to the manufacturer’s instructions. In the first set, we tested 12 leaves from Bt cotton plants (including leaves from all four plants from which bolls yielded AZP-R2 survivors) and 10 leaves from non-Bt cotton plants. In the second set, we tested 18 leaves from Bt cotton plants (including leaves from 15 of 16 plants from which bolls yielded AZP-R2 survivors) and 2 leaves from non-Bt cotton plants. All Bt cotton leaves tested positive for Cry1A and Cry2A (30 of 30) and all non-Bt cotton leaves tested negative for both toxins (12 of 12).

### Data analysis

In diet bioassays, larval survival (%) was calculated as number of fourth instars, pupae, or adults alive at 21 d divided by the initial number of neonates times 100%. For cotton boll assays, we divided survival on two-toxin Bt cotton bolls by the survival on non-Bt cotton bolls (as described above) to calculate the proportional survival on two-toxin Bt cotton relative to non-Bt cotton. We estimated the concentration of toxin killing 50% of larvae (LC_50_) and its 95% fiducial limits from diet bioassays using SAS PROC PROBIT^58^. Resistance ratios were calculated by dividing the concentration of toxin in diet killing 50% of larvae (LC_50_) of a strain by the LC_50_ of the susceptible strain APHIS-S. We estimated dominance (*h*), which varies from zero for completely recessive resistance to one for completely dominant resistance, as previously described^59^.

## Additional Information

**How to cite this article**: Fabrick, J. A. *et al.* Multi-Toxin Resistance Enables Pink Bollworm Survival on Pyramided Bt Cotton. *Sci. Rep.* **5**, 16554; doi: 10.1038/srep16554 (2015).

## Supplementary Material

Supplementary Information

## Figures and Tables

**Table 1:** Six resistant strains and two susceptible strains of pink bollworm tested in this study.

	Toxin(s) selected with	Background	Reference(s)
Resistant			
Bt4R	Cry1Ac	USDA lab strain	33
Bt4R-P	Cry1Ac	Derived from Bt4R	this study
AZP-R	Cry1Ac	Arizona pooled strain	31, 34, 53
Bt4-R2	Cry1Ac, Cry2Ab	Derived from Bt4R	this study
AZP-R2	Cry1Ac, Cry2Ab	AZP-R X Bt4R-2	this study
AZP-R2U	Cry1Ac, Cry2Ab	Derived from AZP-R2, reared on control diet	this study
Susceptible			
APHIS-S	None	USDA lab strain	49
APHIS-SOM	None	APHIS-S × SOM-07^a^	this study

^a^SOM-07 was a susceptible strain derived from the field in Somerton, Arizona in 2007^33^.

**Table 2:** Responses of pink bollworm strains to Bt toxins Cry1Ac and Cry2Ab.

Strain	Date	Gen^a^	n^b^	LC_50_ (95% FL)c[Fn t2-fn3]	RR^d^	Survival (%)^e^
Cry2Ab						
Bt4-R2	March 2012	F_15_	240	5,720 (NA)	150,000	85.7
Bt4R-P	May 2012	F_19_	180	0.0222 (0.013–0.044)	0.58	0.00^f^
Bt4R	May 2012	F_61_	180	0.0273 (0.011–0.038)	0.72	0.00^f^
APHIS-S	May 2012	NA	180	0.0380 (0.014–0.066)	1.0	0.00^f^
Cry1Ac						
Bt4-R2	March 2012	F_15_	240	9.87 (4.4–18)	28	56.5
Bt4R-P	April 2012	F_18_	240	143 (93–200)	400	100
Bt4R	March 2012	F_59_	240	3.79 (1.5–7.1)	11	37.5
APHIS-S	April 2012	NA	180	0.353 (0.094–0.58)	1.0	0.00

^a^Generation tested in bioassays.

^b^Number of larvae tested including controls on untreated diet.

^c^Concentration killing 50% with 95% fiducial limits in parentheses (NA indicates limits not available), in μg of toxin per mL of diet.

^d^Resistance ratio, the LC_50_ for a strain divided by the LC_50_ for APHIS-S (susceptible).

^e^Survival at 10 μg of toxin per mL diet (unless noted otherwise), adjusted for control mortality; sample size for each estimate was 30 neonates.

^f^10 μg Cry2Ab per mL diet was not tested; adjusted survival was 0% at a lower concentration (1 μg Cry2Ab per mL for Bt4R and APHIS-S; 0.3 μg Cry2Ab per mL for Bt4R-P).

**Table 3:** Responses to Bt toxin Cry2Ab of pink bollworm larvae from a resistant strain (Bt4-R2), a susceptible strain (APHIS-S), and their F_1_ progeny.

Strain	n^a^	LC_50_ (95% FL)^b^[Fn t3-fn2]	RR^c^	Survival (%)^d^
Bt4-R2^e^	192	679 (NA)^f^	18,000	75.0
F_1_A^g^	192	0.0780 (NA)	2.0	0.00
F_1_B^h^	192	0.0580 (0.038–0.074)	1.5	0.00
F_1_ pooled	384	0.0620 (0.012–0.078)	1.6	0.00
APHIS-S	192	0.0385 (0.028–0.050)	1.0	0.00

^a^Number of larvae tested including controls on untreated diet.

^b^Concentration killing 50% with 95% fiducial limits in parentheses, in μg Cry2Ab per mL of diet.

^c^Resistance ratio, the LC_50_ for a strain divided by the LC_50_ for APHIS-S (susceptible).

^d^Survival at 10 μg Cry2Ab per mL diet, adjusted for control mortality, n = 64 larvae for F_1_ pooled, 32 for all other estimates.

^e^F_22_ generation of Bt4-R2 was tested in October 2012 and used in crosses with APHIS-S.

^f^Not available.

^g^Male Bt4-R2 X female APHIS-S.

^h^Female Bt4-R2 X male APHIS-S.

**Table 4:** Expected and observed survival of pink bollworm strain AZP-R2^a^.

Generation	Survival (%) vs. Cry2Ab		Survival (%) vs. Cry1Ac	
	Expected^b^	Observed^c^	Expected^d^	Observed^c^
F_1_^d^	0^f^	0.0	100	90
F_2_^e^	25	25	100	100
F_4_	100	97	100	95^g^

^a^AZP-R2 was generated by crossing AZP-R2 with Bt4-R2; F_1_, F_2_, and F_4_ larvae were tested in June, July and August 2012, respectively (see Fig. 1 and text).

^b^Expected survival was calculated based on hypotheses about inheritance of resistance (see text).

^c^Observed survival was measured at the diagnostic concentration of each toxin (10 μg Cry2Ab or Cry1Ac per mL diet), adjusted based on survival on untreated diet (control).

^d^Aside from the larvae used in the bioassays summarized above, the F_1_ larvae were reared on untreated diet.

^e^The F_2_ larvae were selected on diet containing 10 μg Cry2Ab per mL diet.

^f^The sample size for each value of observed survival was 60 to 85 larvae (30 or 40 larvae on treated diet plus 30 to 45 larvae on untreated diet).

^g^For F_1_, F_2_, and F_4_, the observed survival for Cry1Ac (mean = 95%) did not differ significantly from the expected survival (93%) (one-sample t-test, t = 0.69, P = 0.56).
