# Supplementary material for: Multi-Toxin Resistance Enables Pink Bollworm Survival on Pyramided Bt Cotton
Source: Sci Rep. 2015 Nov 12;5:16554. doi: 10.1038/srep16554 (PMC5156061; doi:10.1038/srep16554)
Supplement: Supplementary Information [file srep16554-s1.docx]

**Supplementary Information**

**Multi-Toxin Resistance Enables Pink Bollworm Survival on Pyramided Bt Cotton**

Jeffrey A. Fabrick^1^, Gopalan C. Unnithan^2^, Alex J. Yelich^2^, Ben DeGain^2^, Luke Masson^3^, Jie Zhang^4^, Yves Carrière^2^, and Bruce E. Tabashnik^2^

^1^ USDA ARS, U.S. Arid Land Agricultural Research Center, Maricopa, AZ 85138 USA

^2^ Department of Entomology, University of Arizona, Tucson, AZ 85721 USA

^3^ Biotechnology Research Institute, National Research Council of Canada, Montreal, QC, Canada H4P 2R2

^4^ State Key Laboratory for Biology of Plant Diseases and Insect Pests, Institute of Plant Protection, Chinese Academy of Agricultural Sciences, Beijing, Haidian District, 100193 Peoples Republic of China

**This Supplementary Information contains:**

**Supplementary Table S1**

**Table S1. Survival of pink bollworm on bolls of field-grown cotton.**

Type of Survival on Bt

Strain Gen^a^ cotton^b^ Bolls Larvae Survivors^c^ Survival (%) relative to non-Bt

APHIS-SOM F_55_ Bt 10 150 0 0.00 0.00

APHIS-SOM F_55_ Non-Bt 5 75 12 16.0

AZP-R F_151_ Bt 10 150 0 0.00 0.00

AZP-R F_151_ Non-Bt 5 75 4 5.33

Bt4-R2 F_17_ Bt 10 150 0 0.00 0.00

Bt4-R2 F_17_ Non-Bt 5 75 10 13.3

AZP-R2U ^d^ F_3_ Bt 10 150 0 0.00 0.00

AZP-R2U ^d^ F_3_ Non-Bt 5 75 12 16.0

AZP-R2 F_3_ Bt 10 150 5 3.33 0.17

AZP-R2 F_3_ Non-Bt 5 75 15 20.0

AZP-R2 F_4_^e^ Bt 20 449 25 5.57 0.17

AZP-R2 F_4_^e^ Non-Bt 4 81 26 32.1

^a^ Generation tested on bolls.

^b^ Bt cotton produced Cry1Ac and Cry2Ab

^c^ Live fourth instars, pupae, and adults were scored as survivors.

^d^ A subset of AZP-R2 that was reared without additional selection with Cry2Ab.

^e^ AZP-R2 F_4_ was tested in September 2012, with the initial sample size based on the number of entry holes caused by neonates that emerged from eggs; all others were tested in August 2012 with the sample size based on the number of neonates we placed on bolls (see Methods).
